# Generation of Chimeric Antigen Receptors against Tetraspanin 7

**DOI:** 10.3390/cells12111453

**Published:** 2023-05-23

**Authors:** Tom Pieper, Kristian Daniel Ralph Roth, Viktor Glaser, Tobias Riet, Laura Elisa Buitrago-Molina, Maike Hagedorn, Maren Lieber, Michael Hust, Fatih Noyan, Elmar Jaeckel, Matthias Hardtke-Wolenski

**Affiliations:** 1 Department of Gastroenterology, Hepatology, Infectious Diseases & Endocrinology, Hannover Medical School, 30625 Hannover, Germany; 2 Institut für Biochemie, Biotechnologie und Bioinformatik, Abteilung Medizinische Biotechnologie, Technische Universität Braunschweig, 38106 Braunschweig, Germany; 3 Department I of Internal Medicine, Tumor Genetics, University Hospital of Cologne, Center for Molecular Medicine Cologne (CMMC), University of Cologne, 50933 Cologne, Germany; 4 Department of Liver Transplantation, Multi Organ Transplant Program, University Health Network, University of Toronto, Toronto, ON M5T 0S8, Canada; 5 Institute of Medical Microbiology, University Hospital Essen, University Duisburg-Essen, 47057 Essen, Germany

**Keywords:** chimeric antigen receptor, type 1 diabetes, beta cells, TSPAN7

## Abstract

Adoptive transfer of antigen-specific regulatory T cells (Tregs) has shown promising results in the treatment of autoimmune diseases; however, the use of polyspecific Tregs has limited effects. However, obtaining a sufficient number of antigen-specific Tregs from patients with autoimmune disorders remains challenging. Chimeric antigen receptors (CARs) provide an alternative source of T cells for novel immunotherapies that redirect T cells independently of the MHC. In this study, we aimed to generate antibody-like single-chain variable fragments (scFv) and subsequent CARs against tetraspanin 7 (TSPAN7), a membrane protein highly expressed on the surface of pancreatic beta cells, using phage display technology. We established two methods for generating scFvs against TSPAN7 and other target structures. Moreover, we established novel assays to analyze and quantify their binding abilities. The resulting CARs were functional and activated specifically by the target structure, but could not recognize TSPAN7 on the surface of beta cells. Despite this, this study demonstrates that CAR technology is a powerful tool for generating antigen-specific T cells and provides new approaches for generating functional CARs.

## 1. Introduction

Type 1 diabetes (T1D) is an emerging issue, particularly in the Northern Hemisphere and most developed countries. It is one of the most common autoimmune diseases and mostly affects children and adolescents. T1D destroys insulin-producing beta cells in the pancreas, leading to impaired glycoregulation. The etiology has not yet been completely resolved, but appears to be multifactorial and is based on genetic as well as environmental factors. To date, exogenous insulin administration has been the only effective treatment. Nevertheless, the average lifespan of T1D patients is still reduced by approximately 10 years, and the incidence in the Western world dramatically increases by 2–3% every year [1,2,3,4,5,6].

Autoreactive T cells are drivers of autoimmune diseases such as T1D. Although the onset of T1D is usually preceded by the presence of autoantibodies against characteristic beta cell antigens [7], beta cell degradation is caused by the direct action of beta-cell-specific T cells within pancreatic islets. These autoreactive cells can emerge through defective negative selection, which usually ensures central tolerance of the thymus [8]. Ongoing beta cell death leads to a lack of insulin, and in patients with recent-onset T1D, only 30% of all pancreatic islets are insulin-positive [9]. Nevertheless, functional beta cells can be found in patients, even after 50 years of ongoing diabetes [10]. This phenomenon suggests that therapies that delay or even halt the progressive loss of beta cell mass may be effective in the treatment of diabetes.

Regulatory T cells (Tregs) control autoreactive T cells via peripheral tolerance mechanisms. They can act either directly through inhibitory cytokine secretion, cytolysis, cytokine deprivation, and metabolic disruptions, or indirectly through alterations in dendritic cell function and maturation [11]. An imbalance between autoreactive T cells and Tregs is considered a hallmark of autoimmune disease development [12]. In a non-obese diabetic (NOD) mouse model, altering Treg specificities or binding ameliorated diabetes onset and progression. Monoclonal Tregs expressing an autoreactive transgenic T-cell receptor (TCR) (BDC2.5) efficiently inhibited the development of diabetes at a dose of 50,000 transgenic Tregs and even showed a significant delay in the onset of diabetes when applied at a dose of 500 transgenic Tregs [13,14]. These TCR transgenic cells had a comparable effect when converted from effector T cells (Teffs) to converted Tregs (cTregs) by the recombinant co-expression of FoxP3, the predominant phenotype marker of Tregs [15]. Treg/Teff ratios were improved by combination treatment with the IL-2/anti-IL-2 complex and transgenic TCR-specific peptide/MHC tetramers in vivo. This finding was supported by an increased accumulation of specific Tregs within the pancreatic islets as well as the prevention of spontaneous diabetes onset [16,17].

However, the use of distinct TCR clones is not applicable to patients with T1D because of MHC restrictions. Therefore, various attempts have been made to enrich the endogenous pool of polyspecific Tregs in patients in vitro for use in autologous therapies. Unfortunately, this approach showed no clinical effects in patients with T1D [18].

Chimeric antigen receptor (CAR) T-cells may be a promising option to overcome this challenge. CAR Teffs are an established treatment for hematopoietic cancers such as B-cell lymphoma [19]. Its use in the treatment of solid tumors is currently under clinical investigation [20]. We and others have shown that the combination of CARs and Tregs is a promising approach to suppress allospecific immune responses driven by MHC I proteins in humanized transplantation models [21]. This treatment is currently under ongoing clinical investigation for MHC-mismatched kidneys (NCT04817774) and liver transplantation (NCT05234190).

The pool of endogenous beta-cell-specific Tregs in T1D patients is limited [22,23]. Therefore, CAR Tregs could be an effective approach to redirect endogenous Tregs toward beta cells and reduce autoinflammation in T1D patients. Enrichment and activation of these cells within pancreatic islets could suppress inflammation, inhibit beta cell loss, and even enable cell regeneration, eventually leading to the restoration of insulin production.

We chose the membrane protein tetraspanin 7 (TSPAN7) as the target antigen to generate single-chain variable fragments (scFvs) by phage display, which were used as antigen receptors in CAR constructs. TSPAN7 belongs to the transmembrane 4 superfamily (TM4SF) of proteins and is characterized by four clustered transmembrane domains and a large and short extracellular loop [24]. They play a role in vesicle transport and secretion by interacting with specific proteins. TSPAN7 is expressed in the pancreatic islets and is a target of autoantibodies in T1D patients. As all autoantibody epitopes for TSPAN7 are cytoplasmic, it is unlikely that TSPAN7 is directly involved in beta cell destruction. TSPAN7 likely acts as a trigger for diabetogenic T cells during the upregulation of MHC I antigen presentation [25,26]. Nevertheless, it is abundantly expressed in beta cells within pancreatic islets in humans [27].

Thus, TSPAN7 is a promising target for novel CAR-Treg approaches for the treatment of T1D.

## 2. Materials and Methods

### 2.1. ScFv Generation by Phage Display

ScFvs, which were used as antigen receptors in our CAR constructs, were screened by phage display from human naïve antibody HAL9/10 gene libraries, as previously described [28,29]. Briefly, TSPAN7 was bound to a Nunc MaxiSorp plate (Invitrogen, Aachen, Germany) either by direct coating as a recombinant fusion protein spanning the extracellular loop between aa 113–213 and fused to 6x His Tag (Sino Biologicals, Eschborn, Germany), or by indirect binding to coated streptavidin as a 17 aa peptide spanning aa 113–129 and fused to biotin (Peps4LS). Initially, 5 × 10^11^ phage particles from HAL9/10 libraries packaged with hyperphages were incubated on coated plates. Non-binding phage particles were removed by numerous wash cycles (1–5 depending on the panning round) with Tris-buffered saline with Tween 20 detergent (TBS-T). Bound phages were eluted by incubation with trypsin at 37 °C. Phage particles were reamplified by co-infection with *E. coli* TG1 and the M13K07 helper phage. Panning was performed for up to 5 rounds. After the final round, soluble monoclonal scFv was produced in microtiter plates and further characterized using antigen ELISA, cell binding by flow cytometry, and immunofluorescent staining. DNA sequences of positive candidates were isolated, sequenced, and cloned into two different 2nd generation CD28/CD3ζ CAR scaffolds with either a long Fc- or short CD8-derived hinge.

### 2.2. Antigen ELISA

Nunc MaxiSorp plates (Invitrogen) were coated with either TSPAN7 antigen (see Section 2.1) or bovine serum albumin (BSA) as a control antigen. The plate was blocked with 2% MPBST (2% skim milk powder in PBS with 0.05% Tween-20 (MPBST)) for 1 h and washed three times. The wells were incubated with 100 µL soluble scFv (1:1 diluted with 2% MPBST) for 1.5 h and washed three times. ScFv was bound to the α-Myc-specific mouse antibody 9E10 (sc-40, Santa Cruz Biotechnology, Heidelberg, Germany), followed by incubation with the α-mouse Fc-specific HRP-conjugated antibody (A0168, Sigma-Aldrich, Darmstadt, Deutschland) for 1 h. The staining reaction was initiated by adding 3,3,′5,5′-tetramethylbenzidine (TMB) substrate solution and was stopped by adding 2 N H_2_SO_4_. Absorption was measured using an ELISA Reader at 450/620 nm (TECAN Sunrise).

### 2.3. ScFv Cell Binding in Flow Cytometry

HEK293T cells were transfected with the TSPAN7-P2A-eGFP target constructs or left untreated. After 24 h, the transfection efficiency of cells was measured with GFP staining using FACS. The cells were mixed with untransfected cells to obtain a mixture of approximately 20–50% GFP+ cells. Cells were washed with MACS buffer and seeded in a 96-well V-bottom polystyrene plate (Greiner Bio-One, Frickenhausen, Germany) at 1 × 10^5^ cells/well in 100 µL of MACS buffer. The plate was centrifuged and the cells were resuspended in 60 µL of soluble scFv and incubated for 20 min at room temperature (RT). The plate was washed with 100 µL MACS buffer and centrifuged. Bound scFv was stained with 50 µL of α-His-PE antibody (GG11-8F3.5., Miltenyi Biotec, Bergisch Gladbach, Germany) for 20 min at RT. The cells were analyzed using flow cytometry (Cytoflex S, Beckman Coulter, Krefeld, Germany).

### 2.4. Immunofluorescence Staining of scFv

Paraffin-embedded and formalin-fixed sections of murine and human pancreases were stained for soluble scFv. The myc tag was bound using an anti-myc-tag rabbit antibody (C3956, Sigma-Aldrich) and subsequently stained with goat anti-rabbit Alexa Fluor™ 488 conjugated antibody (A-11034, Invitrogen). Stained sections were embedded in DAPI Fluoromount medium (Invitrogen). Insulin antibody was used as a positive control as previously described [30]. Non-specific scFvs were used as isotype controls. Stained sections were analyzed using an AxioImager M1 microscope and ZEN 3.0 software (Zeiss, Jena, Germany).

### 2.5. Vector Design and Retrovirus Production

Retroviral particles were produced as previously described [31]. Briefly, HEK293 T cells were transfected with CAR constructs encoding scFv connected by either a murine CD8-based hinge (short) or murine Fc-IgG-based hinge (long) to a second-generation CAR signal domain based on murine CD28 and CD3ζ. CAR genes were P2A-separated from an additional FoxP3 expression cassette. All constructs contained Thy1.1, a transduction marker under the control of an internal ribosome entry site (IRES). For a detailed vector design, see corresponding figure. Equal amounts of K37- and Gag/Pol-containing helper plasmids were transfected into cells. The viral particles were harvested after 24 h and concentrated using centrifugation overnight at 10,000× *g* rpm at 4 °C. The viral particles were used at an MOI of 25.

### 2.6. Murine CAR T Cell Activation Assay

Murine splenocytes were isolated from BALB/c mice, and CD4+ Teffs were isolated using a magnetic MojoSort™ mouse CD4 T cell isolation kit (BioLegend, San Diego, CA, USA). CD4 Teffs were activated using a CD3/CD28 murine Treg expansion kit (Miltenyi Biotec) and cultured for 48 h. The cells were transduced with retroviral particles containing CAR vectors by spin transduction at 800× *g* for 1.5 h. 48 h after transduction, CAR T cells were magnetically debeaded from CD3/CD28 activation beads. Cells were stained with carboxyfluorescein succinimidyl ester (CFSE) at 37 °C for 10 min. The cells were washed and 5 × 10^4^ cells were seeded in 96-well culture plates (Greiner Bio-One) into wells previously coated with TSPAN7 or FAM159 peptide as a control antigen or left untreated but containing CD3/CD28 Dynabeads (Invitrogen). For coating, the wells were preincubated for 2 h at 37 °C with 1 µg/mL recombinant TSPAN7 or the control antigen. Cells were incubated at 37 °C for 72 h. Cells were then stained with anti-CD4, anti-CD69, and anti-Thy1.1 antibodies (BioLegend), and cell activation and proliferation were determined using flow cytometry.

### 2.7. Hybridoma Coculture Assay

CAR constructs were tested in vitro in a murine NFAT-GFP hybridoma cell line that encoded GFP under the control of an NFAT-dependent IL-2 promoter [32]. Transduction of the hybridoma cells was performed as described in Section 2.5. HEK293T cells were transfected with the TSPAN7 antigen vector to express the target antigen. TSPAN7 expression was verified with flow cytometry staining with an anti-TSPAN7 Alexa Fluor™ 647-conjugated antibody (FAB9179R, R&D Systems, Wiesbaden, Germany). After 48 h, hybridoma cells were either activated in TSPAN7 protein- or peptide-coated wells, or mixed with antigen-expressing HEK293T cells in 48-well culture plates (677 180, Greiner Bio-One). After 24 h, the cells were harvested and stained with an anti-Fab fragment Alexa Fluor 647-conjugated antibody (109-605-006; Jackson ImmunoResearch, West Grove, PA, USA) for CAR expression and an anti-Her-2 PE-conjugated antibody (324405; BioLegend) for negative gating of HEK293T cells. Cells were analyzed by flow cytometry for the HEK293T marker (PE), CAR expression (Alexa Fluor™ 647), and CAR activation (endogenous GFP).

### 2.8. Data Analysis

The data were analyzed and visualized using FlowJo 10 software (BD Biosciences, Heidelberg, Germany), CytExpert 2 software (Beckman Coulter), and Prism 8 software (GraphPad, San Diego, CA, USA). Schematic workflows were generated using BioRender. *p*-values were determined using two-way ANOVA and multiple comparison testing (Benjamini, Krieger, and Yekutieli post hoc test). * *p* < 0.033, ** *p* < 0.002, *** *p* < 0.001.

## 3. Results

### 3.1. Schematic of Phage Display Used to Generate Single-Chain Fragments against the TSPAN7 Protein

**Figure 1:**
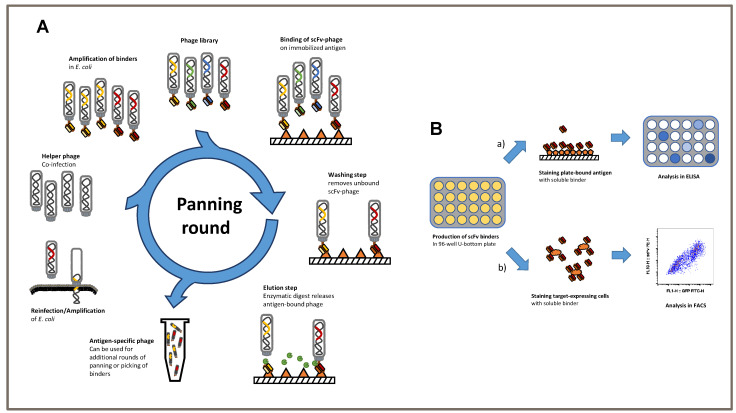
Protein panning as a tool to generate specific scFv used for CARs. (**A**) Overview of panning protocol on plate-bound antigen, showing one complete panning round. (**B**) Alternative ways of screening of binders after protein panning. Screening ELISA shown above. Screening procedure on antigen-transfected cells by flow cytometry shown below.

We established a phage display protocol to produce specific TSPAN7-binding single-chain fragments (scFvs) (Figure 1A). Considering previous approaches, we first performed three consecutive panning rounds on the antigen of interest. The removal of non-specific binding scFvs was performed using BSA in all three rounds. First, scFvs were analyzed using antigen ELISA to verify the specificity of TSPAN7 protein (Figure 1B upper).

### 3.2. Generation of TSPAN7-Specific scFvs

**Figure 2:**
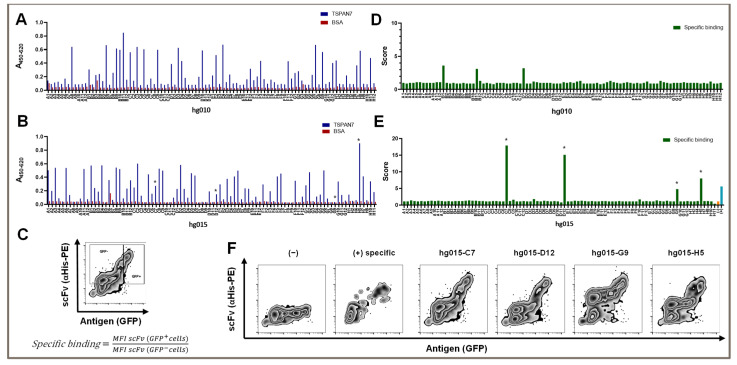
Protein panning yields TSPAN7-specific binders. (**A**) Screening ELISA of 96 scFv binders (hg010 A1-H12) after the 3rd round of protein-based panning. scFv were stained using secondary staining of the appending His tag by anti-His tag antibody and HRP conjugate. (**B**) Screening ELISA of 92 scFv binders (hg015 A1-H11) obtained by the same procedure but after the 5th round. (**C**) Example of scFv binder screening on antigen-transfected HEK293T cells in FACS and score calculation. Cells were stained with soluble scFv hg015-C7 and detected using secondary staining of the appending His tag by PE anti-His tag antibody. TSPAN7 antigen expression was measured using GFP coexpression. Specific binding score was calculated according to the formula below. (**D**) FACS Screening of TSPAN7-transfected HEK293T cells for the binders obtained in (**A**). (**E**) FACS Screening of TSPAN7-transfected HEK293T cells for the binders obtained in B. Positive control of an scFv detecting a transfected control antigen on cells in blue. Medium-only control in orange. (**F**) Representative plots of specific scFv binders hg015-C7, hg015-D12, hg015-G9, and hg015-H5 showing binder TSPAN7-transfected HEK293T cells. Medium control (−) and unrelated specific binder as positive control (+) shown left. Binders are also indicated with asterisks in (**E**).

After the third panning round, we obtained 36 of the 92 screened clones with a suitable signal-to-noise ratio (Figure 2A). In detail, we considered clones with a 450/620 nm absorption signal over 0.2 in TSPAN7 ELISA and a signal under 0.1 in BSA ELISA as hits. The average hit signal was 0.46, with the highest hit (hg010-B11) reaching 0.85. Only two non-specific clones with a BSA signal over 0.1 were found.

We investigated whether conducting two additional panning rounds could increase the outcome of TSPAN7-positive hits or improve signals, as repeated panning rounds might increase the affinity of isolated binders for their target. Interestingly, the number of hits improved only slightly, to 41 positive hits. Furthermore, the average signal remained at the same level (0.42); however, the number of strong hits over 0.6 was significantly reduced to two (nine hits after three panning rounds). Again, there was one strong hit with a signal reaching 0.9 (hg015-H5) (Figure 2B).

In summary, three panning rounds were optimal for generating scFvs against TSPAN7. Additional panning rounds did not improve the number of clones or binding capacity.

### 3.3. TSPAN7-Specific scFv Recognized the Protein in Its Natural Form, as Shown by Our Newly Established Scoring System

The TSPAN7-specific scFv was generated using phage display technology against recombinant TSPAN7. However, for successful use in later experiments, it was essential that the complete antigen be recognized on the cell surface rather than the recombinant native protein. We tested our protein-panning-generated binders on HEK293T cells transfected with TSPAN7, in addition to classic ELISA testing. Moreover, TSPAN7-positive cells co-expressing GFP were stained with soluble scFvs, which were detected by staining the attached His-Tag with a PE-labeled secondary antibody and analyzed by flow cytometry (Figure 1B below and Figure 2C–F).

We established a scoring scheme to quantify binding abilities in this assay (Figure 2C). As a new indicator of specific binding, we divided the individual mean fluorescence intensity (MFI) of the scFv signal of TSPAN7-positive cells by the MFI of the scFv signal in non-transfected cells in the same mix. The theoretical values for non-specific binding and non-binding were approximately 1. We set a minimum value of two to detect binders with a dim signal. This indicated that the MFI signal in TSPAN7-positive cells must be at least twice as strong as that in cells without TSPAN7. As shown in Figure 2D,E, scores for positive scFv binders significantly increased between the third and fifth panning rounds. The fifth round of protein panning generated scFv binders with a score of up to 17. In contrast to the score strength, approximately the same number of positive hits were obtained in the third and fifth panning rounds.

We found that several clones showed favorable and strong binding patterns in flow cytometry after the fifth panning round (Figure 2E). These binders showed various signals in the antigen ELISA, varying from high to no TSPAN7 signal (asterisks in Figure 2B).

However, all binders showed distinct staining in the non-transfected cells. This was also true for clone H5, which yielded the highest signal in the antigen ELISA and had a score of 8 for TSPAN7-positive cells. Partially non-specific binding in non-transfected cells was observed for all positive hits after the fifth round (Figure 2F).

### 3.4. A Peptide Panning-Based scFv Showed Recognition of Cell- and Tissue-Expressed TSPAN7

To further shift our scFv/binder selection toward a more defined part of the extracellular loop, we synthesized a 17 aa long peptide using the first part of the larger extracellular loop (aa 113–129). We hypothesized that this strategy would increase our binder output, and we also considered the homology between human and murine TSPAN7 to be important for the translational potential of CARs. Therefore, we selected a sequence area identical to that of humans and mice.

**Figure 3:**
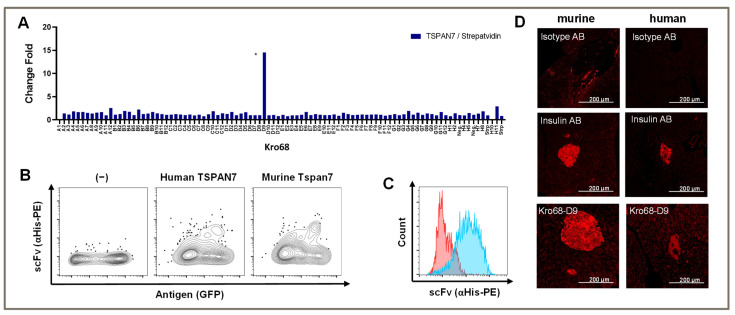
Peptide panning generated a single binder detecting a TSPAN7 peptide. (**A**) Screening ELISA of scFv binders generated by peptide panning. Change fold of A450-620 absorption signals of ELISA on TSPAN7 peptide and streptavidin control antigen. ScFv binder Kro68-D9 indicated with asterisk. (**B**) Flow cytometric staining of Kro68-D9 on TSPAN7-transfected HEK293T cells. ScFv staining with anti-His-PE antibody. TSPAN7 expression reported by GFP. Medium-control left, Kro68-D9 scFv staining on human TSPAN7 and murine TSPAN7-transfected HEK293T cells middle and right, respectively. (**C**) Staining of peptide binder Kro68-D9 on murine beta cell line MIN6. Binding of Kro68-D9 on MIN6 shown in blue. Binding of an unspecific control scFv in red. (**D**) Immunohistochemistry staining on paraffin pancreas sections. Murine and human sections left and right, respectively. First row shows isotype control antibody. Second row shows the positive control with insulin staining, respectively. Below, staining of islets with Kro68-D9 is shown. All pictures 20× magnification.

Again, panning was performed over three consecutive rounds and depletion was performed on streptavidin, which was used to coat the biotinylated peptide. Interestingly, we only obtained a single binder after the third round with a TSPAN7/Streptavidin fold change of 15-fold (Figure 3A). This binder (Kro68-D9) showed the same binding abilities as previous binders in both human and murine homologs of TSPAN7 expressed by HEK293T cells (Figure 3B).

The binding capacity was analyzed in native beta cell lines. We stained the murine beta cell line MIN6 with scFv and analyzed the stained cells with flow cytometry (Figure 3C). Indeed, scFv bound to the beta cell line, whereas an unspecific control scFv did not, indicating the recognition of naturally expressed TSPAN7 in cells.

To further analyze the binding specificity of Kro68-D9 to pancreatic islets, we stained murine and human pancreatic sections with this binder (Figure 3D). Distinct islet staining was observed in both tissues, further confirming species cross-reactivity. Compared to human islets, murine islets showed slightly more intense binding. The staining intensity was comparable to that of insulin, which was used as the positive control in this experiment.

### 3.5. TSPAN7-Specific CAR Can Be Expressed and Stimulated in Murine T Cells

**Figure 4:**
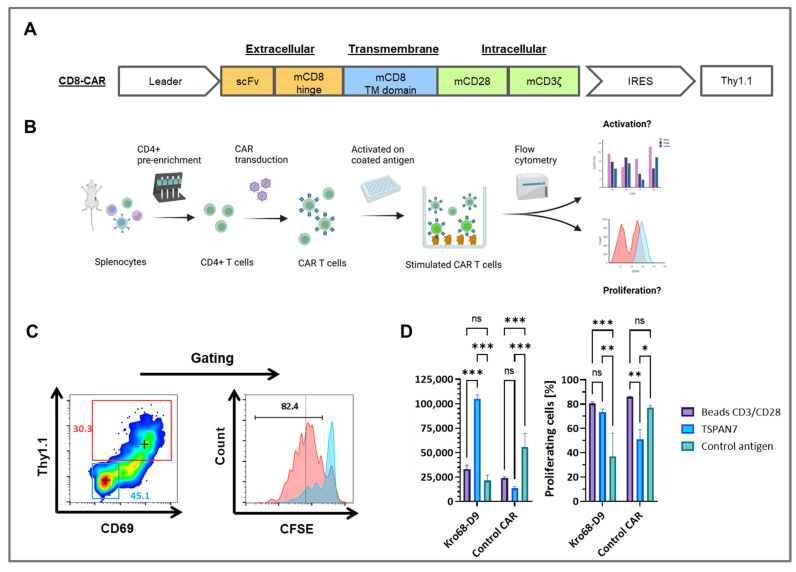
Kro68-D9 CAR T cells are activated by TSPAN7. (**A**) Murine T cells were transduced with 2nd generation short-hinge (CD8-based) CAR containing TSPAN7-specific scFv binder Kro68-D9 and Thy1.1 as reporter gene under control of IRES. (**B**) Schematic overview of CAR T cell activation assay. (**C**) BalbC CD4+ T cells can be efficiently transduced by TSPAN7-specific CAR Kro68-D9. Thy1.1 and CD69 expression is shown for CD4+ pre-gated T cells after stimulation with TSPAN7. Thy1.1+ cells (red gate) show strong proliferation indicated by CFSE dilution compared to untransduced cells (blue gate). Cross indicates MFI within gates. (**D**) Kro68-D9 CAR T cells specifically upregulate CD69 and increased proliferation after stimulation with TSPAN7. Stimulation on CD3/CD28 beads and control antigen is shown. A control CAR specific to the control antigen is shown as a further control. Data are presented as mean ± SD (*n* = 3). *p* values determined by two-way ANOVA and multiple comparison testing (Benjamini, Krieger and Yekutieli post hoc test). * *p* < 0.033, ** *p* < 0.002, *** *p* < 0.001, ns = not significant.

To test whether TSPAN7-specific scFvs function as CAR, Kro68-D9 was cloned into a short-hinge second generation CAR format (Figure 4A). CAR contains a CD8-derived hinge region and the CD28 and CD3ζ costimulatory domains. Thy1.1, serves as a reporter gene and is expressed under the control of an internal ribosomal entry site (IRES). The CAR construct was transduced by gamma-retroviral vectors into CD4+ cells isolated from the splenocytes of BALB/c mice, and the corresponding CAR T cells were activated on plate-bound TSPAN7 protein (Figure 4B). 

Murine CD4+ cells were efficiently transduced with TSPAN7-specific CAR construct Kro68-D9 of up to 30% (Figure 4C). Furthermore, CAR T cells exhibited strong CAR-dependent activation, as indicated by the upregulation of CD69 in the Thy1.1+ fraction. This fraction showed strong proliferation compared to the untransduced cells displayed by CFSE dilution after four days. 

In contrast, CD69 upregulation and proliferation were reduced when Kro68-D9 CAR T cells were cultured on an unrelated control antigen instead of TSPAN7 (Figure 4D). CAR directed against the control antigen, previously developed by our group, showed better activation and proliferation on the control antigen than TSPAN7. Interestingly, CD69 upregulation by the target protein exceeded that observed upon non-specific stimulation with the CD3/CD28 beads. This might be due to the harsher and faster activation of T cells by CD3/CD28 beads, resulting in decreased levels of the early activation marker CD69 after four days. However, the percentage of proliferated cells was comparable after four days for Kro68-D9 CAR T cells in both conditions. Interestingly, a profound proliferative background was observed when cells were cultured on the control antigen. This might be due to strong activation during the initial generation of CAR T cells. 

These findings demonstrated the efficient transduction of TSPAN7-specific CAR into murine T cells and CAR-dependent activation of the cognate target TSPAN7.

### 3.6. TSPAN7-Specific CARs Can Be Activated by Peptides or Proteins but Not Cell Surface-Expressed TSPAN7

**Figure 5:**
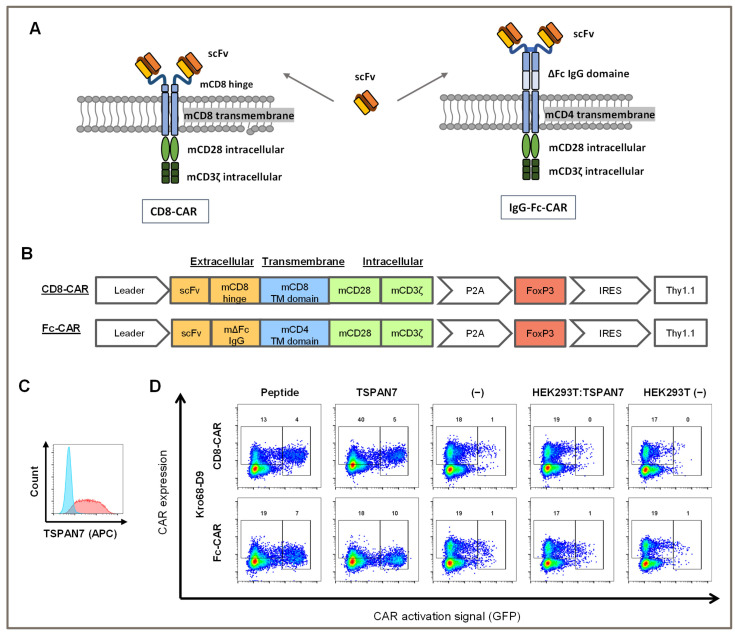
Kro68-D9 CAR can detect TSPAN7 protein and peptide but not surface-expressed TSPAN7 on cells. (**A**) 2nd generation CAR constructs used for cloning of scFvs. Short-hinge CAR shown left and long-hinge CAR right, respectively. (**B**) Schematic overview of used CAR construct—short- and long-hinge CAR, respectively. (**C**) TSPAN7 can be stained on the surface of transfected HEK293T cells. Anti-TSPAN7 antibody staining on untransfected (blue) and transfected cells (red). (**D**) CAR activation in murine hybridoma cells following stimulation by target. NFAT activation is reported by GFP and anti-Fab antibody was used for CAR staining. Activation patterns are shown for short-hinge (above) and long-hinge (below) CARs. CARs were tested with TSPAN7 peptide used for panning, TSPAN7 protein, medium (control), HEK293T transfected with human TSPAN7 and untransfected HEK293T (control).

To further investigate the activating potential of TSPAN7-specific scFvs, Kro68-D9 cells were tested in CAR format (Figure 5A,B). In addition to the shorter-hinge CAR (CD8-CAR), Kro68-D9 was cloned into a longer-hinge CAR (Fc-CAR) consisting of an extracellular IgG Fc-derived hinge after scFv, a CD4-derived transmembrane domain, and a CD28/CD3ζ-based signal domain.

These CAR constructs were transduced into a murine NFAT GFP reporter T cell line that encodes GFP under the control of the NFAT-dependent IL-2 promoter. Upon activation of cells due to CAR stimulation by a cognate target structure, upregulation of NFAT leads to the expression of GFP. Therefore, CAR-transduced cells were co-cultured with immobilized peptides and proteins (Figure 5D). They were further tested on HEK293T cells transfected with human TSPAN7 or left untransfected to check for the activation on cell-expressed TSPAN7. Transfection efficiency of HEK293T cells was examined with FACS using a commercial TSPAN7 antibody (Figure 5C). TSPAN7 was abundantly expressed with a transfection efficiency of 89% within those cells.

CAR transduction efficiency for both constructs was 20%, as indicated by CAR staining in the negative controls (medium- and non-transfected HEK293T cells). Autoactivation was minimal in 1% of the GFP-positive cells. In contrast, both the target peptide and protein activated CAR constructs. Interestingly, for the long-hinge CAR, activation by the protein was slightly stronger than that with the peptide, with 10% and 7% of cells being activated, respectively. Activation of the longer-hinge CAR was also more effective than that of the shorter-hinge CAR, as only 4% and 5% of the cells were activated on peptides and proteins, respectively.

In contrast, CAR was not stimulated by HEK293T cells transfected with TSPAN7. Activation remained the same as that in the negative controls and was not above 1%. Concomitantly, CAR expression was not altered, indicating that TSPAN7 expressed in HEK293T cells was ineffective at stimulating CAR.

In conclusion, CD8+ and Fc-CARs generated by scFv against TSPAN7 were activated by peptides or proteins but not by TSPAN7 expressed in HEK293T cells.

## 4. Discussion

In this study, we describe the successful generation of scFv-based CARs by phage display against the beta cell antigen TSPAN7. ScFvs were generated using a protein- and peptide-targeting panning approach and were able to detect the target structures in vitro as native domains as well as expressed in HEK293T cells. CARs that generate CAR-cTregs can be activated using a highly sensitive activation assay.

Surprisingly, the generated TSPAN7 scFv binders showed a different binding pattern on cells than most of the phage-display-generated scFv binders, as previously described [33,34]. For Kro68-D9, instead of staining all transfected cells, only a subpopulation of transfected cells was detected using the scFv. This finding suggested that a subpopulation of antigen-positive cells remained unbound by scFv. Various factors must be considered to explain this phenomenon.

TSPAN7 is a membrane protein that is abundantly expressed on the surface of beta cells. It is a small but complex protein consisting of four transmembrane domains clustered together within the cell membrane [24]. The short loops between these domains, especially the first extracellular (16 aa) and intracellular (11 aa) loops, may induce a high tension within the entire molecule. This tension is absent if the loops are expressed separately as recombinant proteins or as small stretches of peptides. Therefore, the tertiary structure of the protein may be altered, hampering the detection of the full-length target expressed in cells. This notion was previously hypothesized for TSPAN7 by McLaughlin et al. [25]. Moreover, this hypothesis explains the successful detection of pancreatic islets by scFv, as epitopes are often unfolded during the fixation process in IHC.

For some members of the tetraspanin superfamily, disulfide bonds between structural cysteines are crucial for correct folding of the large extracellular domain [35]. Glycosylation of large extracellular domains has also been described [36]. Although the protein was produced in human host cells, it was not possible to ensure that these post-translational modifications were retained during recombinant production, adding another obstacle to the recognition of cell-surface-expressed antigens. Moreover, protein folding underlies complex cellular mechanisms within the cell. This is particularly true for membrane proteins that are translated into the rough endoplasmic reticulum and undergo various modifications, such as phosphorylation, glycosylation, and alteration of functional groups during transport to the cell surface through the Golgi apparatus. Folding is driven by entropy but can also be maintained by chaperones [37,38]. Although misfolding can often be detected and resolved, this mechanism remains error prone. This phenomenon was particularly true for overexpression in HEK293T cells. It cannot be ruled out that alternatively folded forms of TSPAN7 can be expressed on the cell surface, but only particular folded or misfolded forms can be detected by recombinant protein-panning-generated scFv. This heterogeneous expression may explain the distinct staining patterns observed in TSPAN7-expressing HEK293T cells. Additionally, it is possible that epitopes are not accessible on the cell surface. Both antigen targets for panning, the fusion protein and peptide, represent stretches located at the beginning of the loop of the large extracellular domain. Overexpression-driven shedding exposes surface molecules to a subpopulation of HEK293T cells, allowing scFv binding. However, the reason for shedding and its effect on only a subpopulation of cells remain unclear.

Moreover, other membrane proteins expressed in HEK293T cells may also block TSPAN7 binding. For tetraspanins, a significant variance in possible interaction partners was observed. These interactions include protein–protein interactions and the shedding of adhesion molecules. Most interactions occur in the tetraspanin-enriched microdomains (TEMs) [39]. This web of interaction partners is highly variable and may be affected by recombinant overexpression of TSPAN7 on the surface of HEK293T cells. These arguments are supported by the observation that the murine beta cell line MIN6 was successfully detected using the TSPAN7 binder, Kro68-D9. Although this cell line does not represent a completely functional beta cell [40], its expression profile is more representative of natural beta cells than overexpression-driven HEK293T cells. It would be interesting to examine other beta cell lines or single-cell-degraded islets for positive staining with this scFv binder.

Kro68-D9 was successfully incorporated into a second generation CAR vector as a binding domain. In murine CAR T cells, stimulation with TSPAN7 led to enhanced proliferation of CAR-equipped cells, similar to the non-specific stimulation of TCR by CD3/CD28 beads. Increased target-specific proliferation is a key feature of activated CAR-T effectors and Treg cells, allowing amplification of the immune response [21,30,41,42]. However, activation of murine CAR T cells on HEK293T-expressed TSPAN7 could not be tested in this assay, as alloreactive stimulation by HEK239T cells exceeded target-specific stimulation by surface-expressed TSPAN7.

Indeed, in the NFAT reporter assay, TSPAN7 peptide-directed Kro68-D9 showed favorable expression in a short- and long-hinge CAR format and was activated by the peptide and recombinant-expressed TSPAN7 protein. The activation of both target structures was similar, indicating an unaltered form of the epitope in the peptide and recombinant-expressed protein. However, the activation was better in the long-hinge CAR format. This might be due to the correct distance between CAR and its cognate target to form an immunological synapse [43]. Others have suggested that long hinges are superior to short hinges in the recognition of surfaces near epitopes [44,45,46]. As TSPAN7 is a relatively small protein, the epitopes are likely to be located close to the cell surface, promoting advanced activation of a long-hinge CAR.

Although scFv binders could detect TSPAN7 in HEK293T and MIN6 cells, no activation signal was observed for corresponding CARs in HEK293T cells expressing TSPAN7. There may be several reasons for this finding. We have shown that TSPAN7 was expressed in the cells not only by staining with our self-generated scFv, but also by staining with a commercial antibody. However, antigen expression density remains difficult to predict. We and others have previously shown that efficient cross-linking of CARs is key for their activation [30,47]. Therefore, close proximity of target antigens might be needed to trigger the clustering of CAR domains. Other tetraspanin proteins provide this proximity by clustering in the TEMs [39]. Hence, the lack of efficient CAR cross-linking remains unclear. The overexpression of TSPAN7 may have influenced the formation of these clusters in this study.

As mentioned previously, TSPAN7 is a relatively small protein. The fact that neither the short- nor long-hinge CAR effectively recognized surface-expressed TSPAN7 raises the question of whether the epitope is hindered by the cell membrane at the target site [44,48]. For example, a cognate epitope can be buried in the glycocalyx. It remains unclear whether this is a general issue of TSPAN7 expression, or if it is only true for expression in HEK293T cells.

## 5. Conclusions

In summary, the promising target protein TSPAN7 was not suitable for recognition by CARs on the cell surface. In this study, we established two methods for generating scFvs against TSPAN7 and other target structures. Moreover, we established assays to analyze and quantify their binding abilities. Finally, the corresponding CARs are functional and can be activated.

## Data Availability

The data presented in this study are available upon request from the corresponding author.
